# Insights into additional lactone-based signaling circuits in *Streptomyces*: existence of acyl-homoserine lactones and LuxI/LuxR homologs in six *Streptomyces* species

**DOI:** 10.3389/fmicb.2024.1342637

**Published:** 2024-02-08

**Authors:** Amir Salehi-Najafabadi, Sepand Tehrani Fateh, Ghasem Amoabediny, Javad Hamedi

**Affiliations:** ^1^Department of Microbial Biotechnology, School of Biology and Center of Excellence in Phylogeny of Living Organisms, College of Science, University of Tehran, Tehran, Iran; ^2^Research Center for New Technologies in Life Science Engineering, University of Tehran, Tehran, Iran; ^3^School of Medicine, Shahid Beheshti University of Medical Sciences, Tehran, Iran; ^4^Faculty of Chemical Engineering, College of Engineering, University of Tehran, Tehran, Iran

**Keywords:** *Streptomyces*, acyl-homoserine lactone, gamma-butyrolactone, quorum sensing, differentiation, autoinducer

## Abstract

Acyl-homoserine lactones (AHLs), mediating pivotal physiological activities through quorum sensing (QS), have conventionally been considered limited to Gram-negative bacteria. However, few reports on the existence of AHLs in Gram-positive bacteria have questioned this conception. *Streptomyces*, as Gram-positive bacteria already utilizing a lactone-based QS molecule (i.e., gamma-butyrolactones), are yet to be explored for producing AHLs, considering their metabolic capacity and physiological distinction. In this regard, our study examined the potential production of AHLs within *Streptomyces* by deploying HPLC-MS/MS methods, which resulted in the discovery of multiple AHL productions by *S. griseus*, *S. lavendulae* FRI-5, *S. clavuligerus*, *S. nodosus*, *S. lividans*, and *S. coelicolor* A3(2). Each of these *Streptomyces* species possesses a combination of AHLs of different size ranges, possibly due to their distinct properties and regulatory roles. In light of additional lactone molecules, we further confirm that AHL- and GBL-synthases (i.e., LuxI and AfsA enzyme families, respectively) and their receptors (i.e., LuxR and ArpA) are evolutionarily distinct. To this end, we searched for the components of the AHL signaling circuit, i.e., AHL synthases and receptors, in the *Streptomyces* genus, and we have identified multiple potential LuxI and LuxR homologs in all 2,336 *Streptomyces* species included in this study. The 6 *Streptomyces* of interest in this study also had at least 4 LuxI homologs and 97 LuxR homologs. In conclusion, AHLs and associated gene regulatory systems could be more widespread within the prokaryotic realm than previously believed, potentially contributing to the control of secondary metabolites (e.g., antibiotics) and their complex life cycle, which leads to substantial industrial and clinical applications.

## 1 Introduction

**Figure 1:**
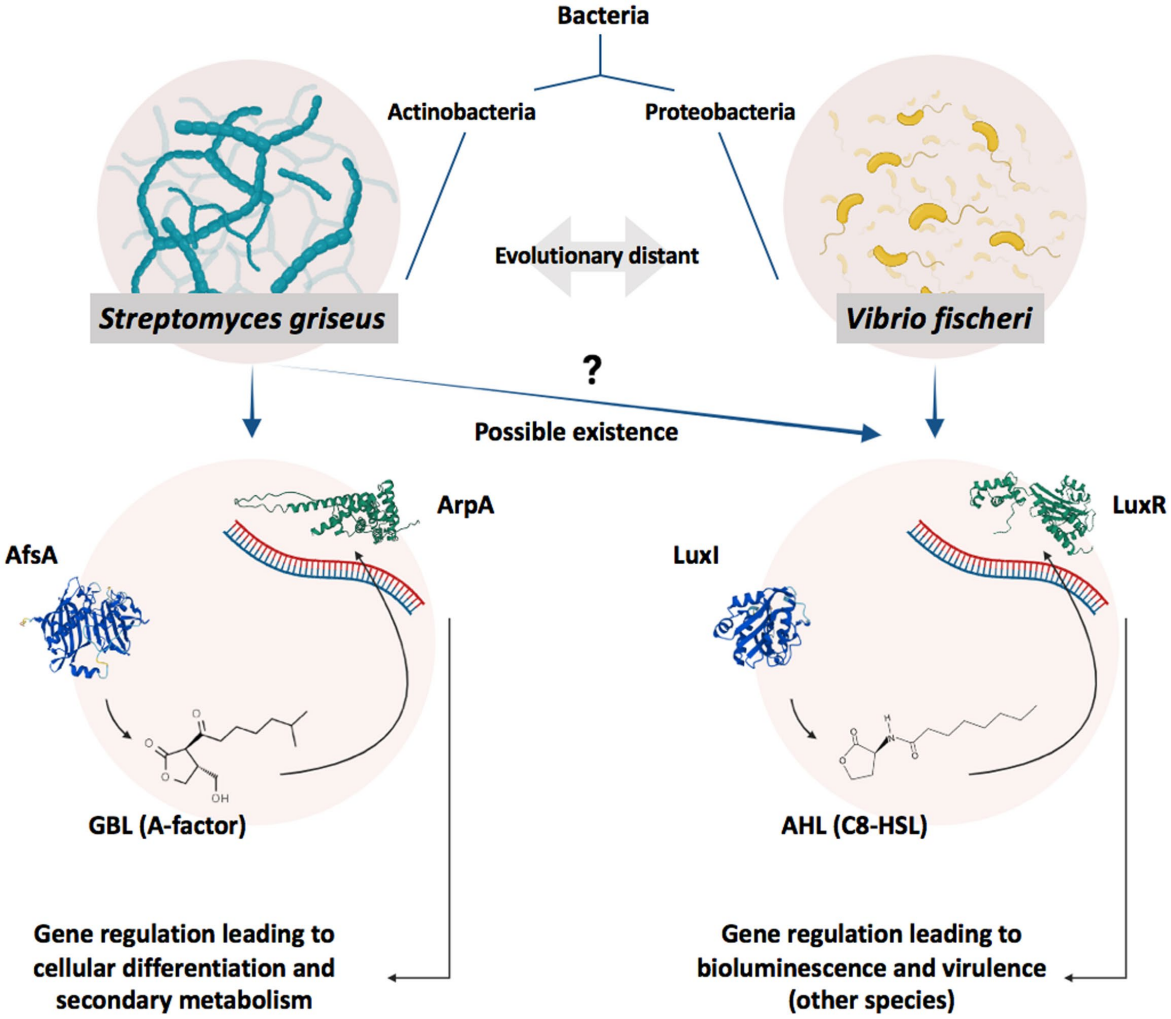
Schematic illustration of GBL- and AHL-based gene regulation systems. GBLs and AHLs, produced by AfsA and LuxI, act as autoinducers in *Actinobacteria* and *Proteobacteria* to regulate the expression of specific genes by affecting their relevant receptors, ArpA and LuxR, respectively, when reaching a specific concentration (reflection of the cell density/population) in a process known as QS (Polkade et al., 2016). A specific level of bacterial population and, subsequently, autoinducer concentration in *V. fischeri* leads to bioluminescence (Engebrecht et al., 1983), while in *Streptomyces* species, it activates cellular differentiation or antibiotic production (Takano, 2006). Bacterial species possessing AfsA/ArpA and LuxI/LuxR protein families are evolutionary distant; however, AfsA and ArpA protein families are considered analogs to LuxI and LuxR protein families, respectively. Regarding the signaling molecules, GBLs and AHLs share structural similarities. Both molecule families possess a gamma-lactone moiety combined with a tail of the carbon chain; however, GBLs usually possess an extra-short branch on the lactone ring while missing the amide bond in the carbon tail. It is worth noting that more structurally different GBLs (Misaki et al., 2022) and AHLs (Schaefer et al., 2008) have also been discovered while all sharing the lactone moiety.

*Streptomyces* species, as filamentous Gram-positive bacteria, are acknowledged for their advanced secondary metabolism and cellular differentiation entangled with complex regulatory circuitry, which revolves around hormone-like signaling molecules known as gamma-butyrolactones (GBLs) (Khokhlov et al., 1967; Horinouchi and Beppu, 1994; Kong et al., 2019; Jose et al., 2021). GBL-based regulatory circuits follow quorum sensing (QS) principles of cooperative population-dependent gene expression and share an architecture with QS systems based on acyl-homoserine lactones (abbreviated as Acyl-HSLs or AHLs); hence, they are considered to be analogous to the latter (Figure 1; Polkade et al., 2016; Barreales et al., 2020).

AHLs were primarily extracted from Gram-negative bacteria as autoinducers running QS gene regulation circuitry (Eberhard et al., 1981). Historically, extensive reports on the existence of AHLs in various Gram-negative bacterial taxa, such as *Proteobacteria* (Fuqua and Greenberg, 2002), *Cyanobacteria* (Sharif et al., 2008), and *Bacteroidetes* (Huang et al., 2008; Twigg et al., 2014), have led to the general assumption that AHLs and related cellular components (i.e., synthases and receptors) are exclusive to these species (Swift et al., 1994; Parsek and Greenberg, 2000; Miller and Bassler, 2001). However, three noticeable studies (Biswa and Doble, 2013; Bose et al., 2017; de Moura et al., 2021) that reported the production of AHLs in some Gram-positive bacteria undermine this broadly accepted view. Following these findings, the discovery of AHL synthases and receptors’ homologs in a limited number of *Streptomyces* species further justifies the quest for AHLs in these species (Santos et al., 2012). Moreover, due to the industrial and medical significance of *Streptomyces* (Khushboo et al., 2022), studying lactone-based signaling circuits and molecules, their evolutionary origin, and the existence of additional relevant circuits and molecules (e.g., AHLs and related circuits) would be of importance. Furthermore, the presence of novel gene regulation mechanisms in *Streptomyces* as complex prokaryotes demonstrating multicellular behaviors (see Flärdh and Buttner, 2009) will be notable from an evolutionary point of view.

In this study, we have demonstrated the production of AHLs by six well-known *Streptomyces* species, including *S. griseus*, *S. lavendulae* FRI-5, *S. clavuligerus*, *S. nodosus*, *S. lividans*, and *S. coelicolor* A3(2), via the chromatography/mass-spectrometry method. Afterward, we investigated the presence of AHL synthases homologous to the LuxI enzyme family and AHL receptors homologous to the LuxR receptor family in *Streptomyces* via *in silico* methods.

## 2 Materials and methods

### 2.1 Culture conditions and preparation of crude extracts

*S. clavuligerus* DSM 738, *S. griseus* DSM 40236, and *S. nodosus* DSM 40109, all obtained from Deutsche Sammlung von Mikroorganismen und Zellkulturen, Germany, *S. lavendulae* FRI-5 MAFF 116015, and *S. lividans* TK-64 MAFF 304033, obtained from the National Food Research Institute, Tsukuba, Japan, and *S. coelicolor* A3(2), obtained from the John Innes Centre, UK, were used as standard strains in all experiments. The strains were cultured on ISP2 agar as sporulation medium consisting of (g/L): malt extract 10, yeast extract 4, glucose 4, CaCO_3_ 2, and agar 18, at pH 7 ± 0.1, and incubated for 8–14 days at 28°C. The same medium was also used as the seeding and fermentation medium after excluding CaCO_3_ and agar. Using ventilation flasks under the mentioned operating conditions prepares the aeration rate of 1 vvm, preventing oxygen limitation during microorganism growth. To this end, for seed culture, ventilation flasks type F1 (modified 250-Erlenmeyer) (Amoabediny and Büchs, 2007) containing 25 mL of ISP2 medium were inoculated with 0.1 mL of spore suspensions of each strain (*ca.* 10^7^–10^8^ spores/ml) and incubated for 72 h, at 28°C and agitated at 220 rpm. The seed culture was then inoculated (5% v/v) into identical ventilation flasks containing 25 mL of fermentation medium and incubated in the same growth conditions as the previous step. After cultivation, the fermentation broth of each strain was acidified to pH 3 by concentrated HCl and centrifuged at 6000 *g* for 15 min. The supernatant was extracted twice with a 5-fold volume of ethyl acetate. For each strain, 3 liters of fermentation medium were used to prepare the crude extract. The solvent layer was evaporated to dryness, re-dissolved in 200 μL of methanol, filtered via 0.22 μm Nylon syringe filters, and finally subjected to chromatography and HPLC-MS/MS analysis. The fresh medium was also extracted using the same method and used as the control group.

### 2.2 Chromatography/mass-spectrometry analysis

HPLC-MS/MS setup consisted of an Agilent ZORBAX HPLC system (SB-C18 column) coupled with an Agilent G6410 triple quadrupole mass spectrometer and an ESI interface. The adjustments of the HPLC setup and the protocol were as follows: a sonicated, degassed, and filtered mobile phase of Acetonitrile:Water (0.1% Formic Acid) consists of 20 min 45% acetonitrile, 90% acetonitrile for 3 min, 7 min 100% acetonitrile, 10 min 45% acetonitrile with a flow rate of 0.6 mL/min. The column temperature was set at 25°C, and 20 μL of the sample was subjected to the instrument via an auto-sampler. Mass spectrometer adjustments were as follows: capillary voltage: 4500 V, mass range: 120–450 amu, Dwell time: 500 ms, fragmentor voltage: 120 V, collision energy: 7. Nine ultra-pure AHLs, including N-butanoyl-L-homoserine lactone (C4-HSL), N-hexanoyl-L-homoserine lactone (C6-HSL), N-(3-oxo-hexanoyl)-L-homoserine lactone (Oxo-C6-HSL), N-heptanoyl-L-homoserine lactone (C7-HSL), N-octanoyl-L-homoserine lactone (C8-HSL), N-(3-oxo-octanoyl)-L-homoserine lactone (Oxo-C8-HSL), N-decanoyl-L-homoserine lactone (C10-HSL), N-dodecanoyl-L-homoserine lactone (C12-HSL), and N-(3-oxo-dodecanoyl)-L-homoserine lactone (Oxo-C12-HSL), purchased from Sigma-Aldrich, were used as standards to determine the characteristic daughter ions, which were found to be at m/z 102, reflecting the lactone ring, similar to previous reports (Morin et al., 2003; Fekete et al., 2007; Bose et al., 2017). Accordingly, each sample was evaluated by the precursor ion method screening for AHL-characteristic daughter ion at m/z 102. Afterward, the total ion chromatogram (TIC) of each bacterial medium extract was screened for major masses, and each mass was depicted independently through an extraction chromatogram (EIC). To further confirm each mass to be an AHL, the concurrent existence of at least one other daughter ion at m/z 56, 74, or 84 was evaluated via the production method (Rodrigues et al., 2022). All the results were analyzed using Agilent MassHunter Qualitative Analysis.

### 2.3 *In silico* studies

#### 2.3.1 Comparison of the model protein components of AHL- and GBL-signaling circuits

The molecular function ontology of LuxI from *V. fischeri* and AfsA from *S. griseus*, as the most investigated AHL- and GBL-synthases model proteins, and LuxR from *V. fischeri* and ArpA from *S. griseus* as the model receptors of AHL and GBL were retrieved from Gene Ontology (GO^1^, Ashburner et al., 2000). The enzymatic reactions of LuxI and AfsA were obtained from Rhea^2^ (Alcántara et al., 2012). Three-dimensional models of the proteins were created using the AlphaFold engine (Jumper et al., 2021; Varadi et al., 2022) and retrieved from EMBL-EBI.^3^

To obtain the conserved regions and residues, LuxI (Accession Number: P12747) and LuxR (Accession Number: P12746) of *V. fischeri* and AfsA (Accession Number: P18394) and ArpA (Accession Number: Q9ZN78) of *S. griseus*, in addition to nine homologs of each protein, were retrieved from the UniProt database. Multiple sequence alignment (MSA) of the retrieved sequences was performed using the ClustalW algorithm (Thompson et al., 1994) on the MEGAx platform (Tamura et al., 2021). Jalview (Version 2, Waterhouse et al., 2009) was used for illustrative purposes and further analysis of the aligned sequences. The molecular function ontology, enzymatic reactions, three-dimensional structure, protein sequence, and conserved regions of these proteins were compared to evaluate their similarity and status of homology.

#### 2.3.2 Investigating the existence of LuxI and LuxR homologs in *Streptomyces*

LuxI and LuxR from *V. fischeri* as model proteins were checked against the non-redundant protein sequence database of *Streptomyces* via protein–protein BLAST (default parameters) to assess the existence of potential LuxI and LuxR homologs in this genus. In order to further evaluate the existence of AHL synthases and receptors, a search for these proteins based on protein models was also conducted. InterPro and KEGG databases were searched for the relevant Hidden Markov Models (HMMs) for AHL synthases homologous to LuxI and AHL receptors homologous to LuxR. HMMs were evaluated for their specificity, and only the specific models were used for further analysis (Supplementary material 1). HMMs for AHL synthases and AHL receptors were concatenated independently. A total of 3,313 *Streptomyces* species genomes curated in RefSeq were retrieved from the NCBI Genome database.^4^ Nucleotide sequences were converted into amino acid sequences using the Prodigal program. HMMs were searched against the sequence database locally using the HMMsearch program. The data were filtered for *E-value* <10^−5^ (significant hits), and in the case of the recognition of a single target by multiple models, the hit with a lower *E*-value was selected. Duplicate species were eliminated based on the highest values of LuxI and LuxR copy number multiplication, which resulted in 2336 unique species. It is worth noting that *S. lividans* TK64 genome did not exist in the *Streptomyces* genome database and *S. lividans* TK24, a close strain, was selected for further in-silico analyses to investigate the presence of LuxI and LuxR homologs.

## 3 Results

### 3.1 *Streptomyces* species produce AHL

**Figure 2:**
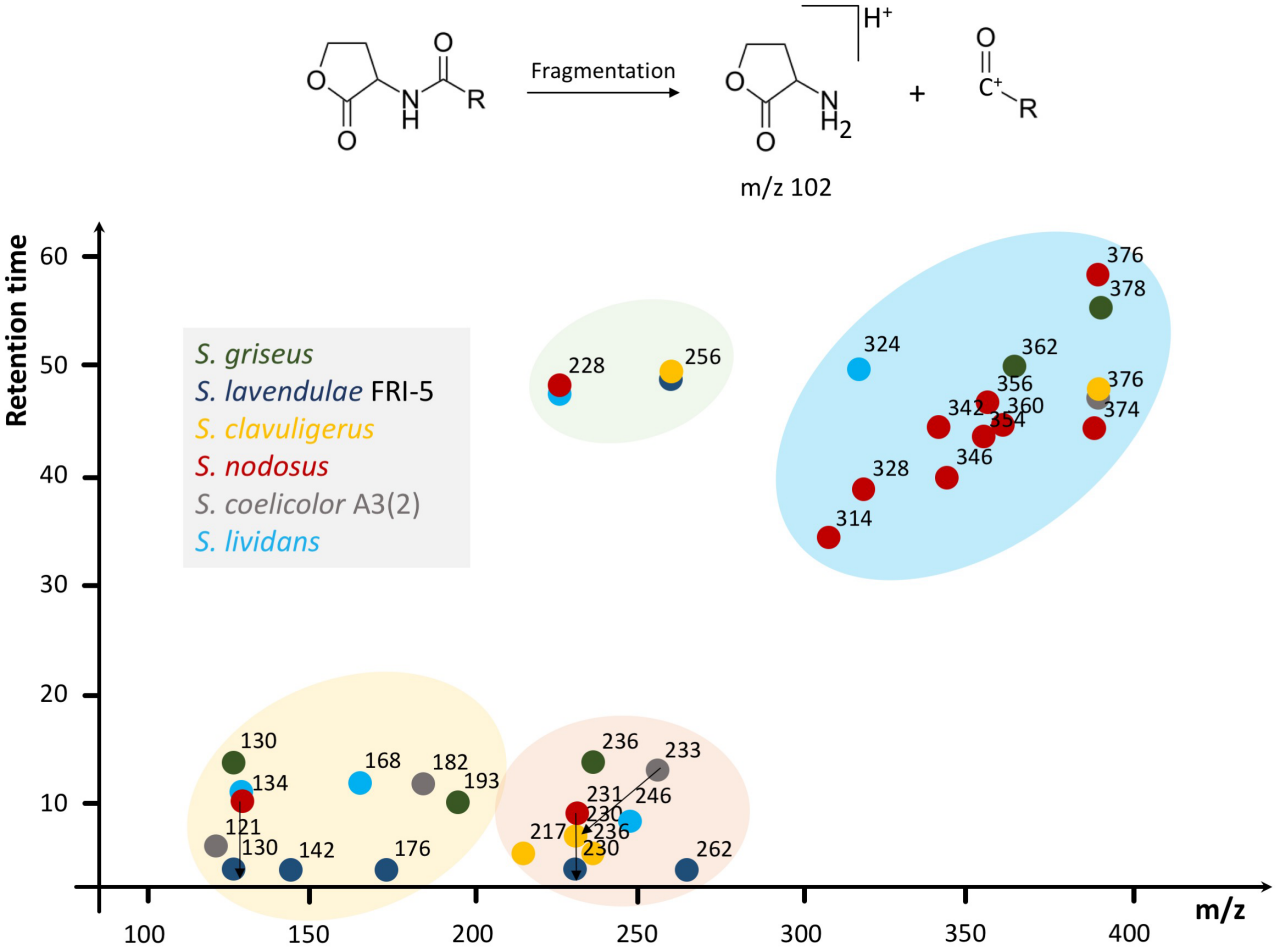
Schematic illustration of various AHLs found in six *Streptomyces* species. Multiple compounds found in *Streptomyces* species via HPLC-MS/MS are summarized based on m/z and retention time in a dot-plot and then clustered according to the parameters to depict the AHL distribution and prevalence regarding their properties. In this regard, four different clusters have appeared. *S. lavendulae* FRI-5 and *S. clavuligerus* do not participate in the yellow and blue clusters, respectively. Furthermore, the green cluster, containing m/z 228 and 256, lacks components from *S. griseus* and *S. coelicolor* A3(2). In addition, 3 m/z ranges of 100–200, 200–300, and 300–400 are evident, which contain AHLs from at least five *Streptomyces* species considered in this study. Original EICs are provided in Supplementary material 2. Different species of *Streptomyces* are represented by colors.

By analyzing the standard AHLs, the characteristic daughter ion of this molecular family was determined to be m/z 102. Afterward, the crude extracts of *Streptomyces* were primarily analyzed for the existence of AHLs by the characteristic m/z 102 daughter ion and then confirmed by other possible daughter ions. Various AHLs were found to be produced by the six *Streptomyces* of interest in this study (Figure 2). In this regard, by considering the significant peaks and comparison of EICs between samples and the control group, the profile of AHL (M + H)^+^ produced by each *Streptomyces* was as follows: *S. griseus*: m/z 130, 193, 236, 362, and 378, *S. lavendulae* FRI-5: m/z 130, 142, 176, 230, 256, and 262, *S. clavuligerus*: m/z 230, 256, and 376, *S. nodosus*: m/z 134, 228, 231, 314, 328, 342, 346, 354, 356, 360, 374, and 376, *S. coelicolor* A3(2): m/z 121, 182, 233, and 376, and *S. lividans* TK-64: m/z 134, 168, 228, 246, and 324. These compounds were further confirmed by the concurrent existence of at least one daughter ion at m/z 56, 74, or 84 via a production method.

### 3.2 LuxI and AfsA enzyme families are not homologs

**Figure 3:**
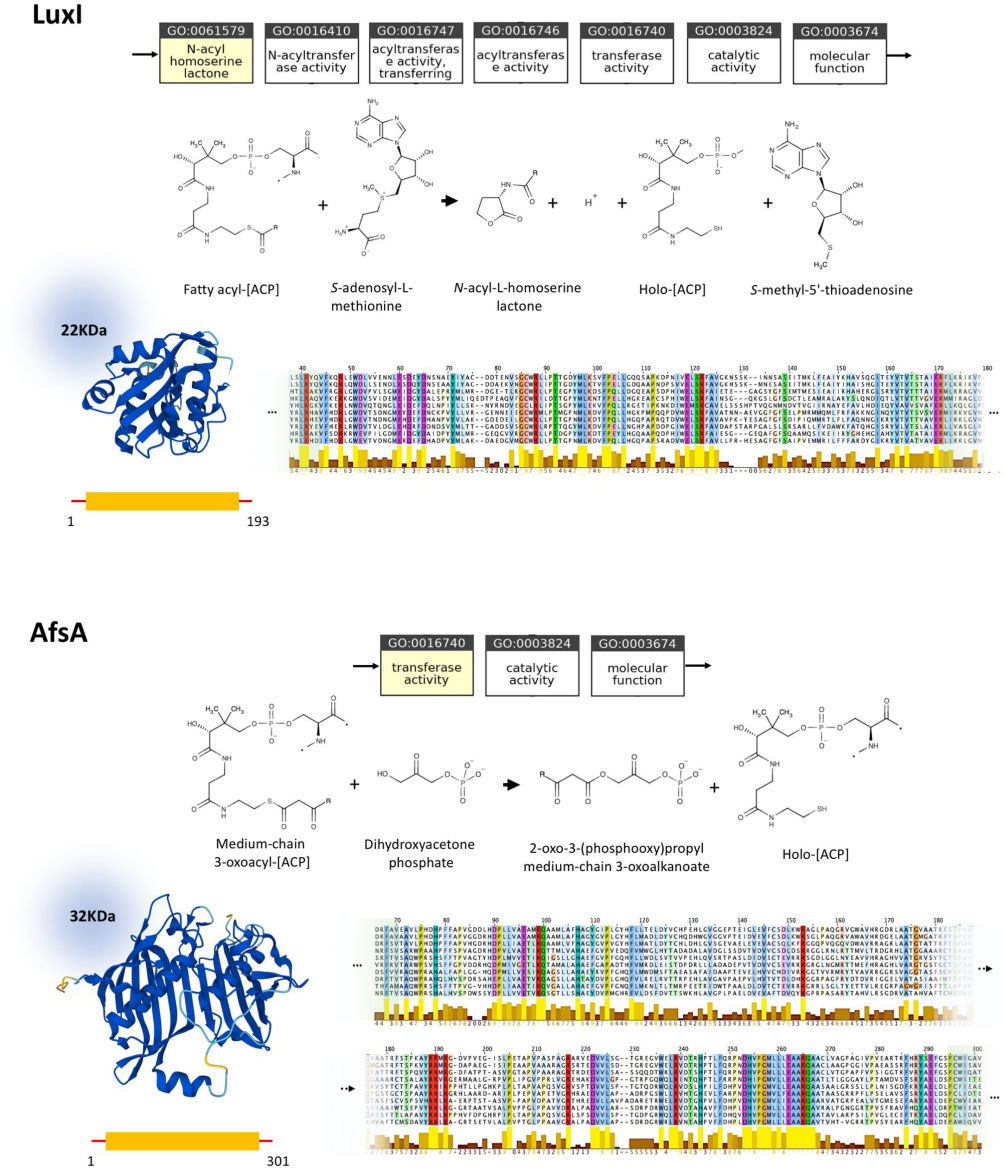
Comparison of LuxI (P12747) and AfsA (P18394) and their homologs. Molecular function ontology, catalytic activity, physical properties, and conserved regions of LuxI and AfsA are demonstrated at the top and bottom, respectively. Both LuxI and AfsA possess transferase activity and share fatty-acid-[ACP] as a substrate, while having a different second substrate. Moreover, these proteins are different in size and topology, as evident in their molecular weight, amino acid sequence length, and three-dimensional structure. LuxI and AfsA enzyme families possess different conserved residues and regions, according to the MSA. The complete MSA is provided in Supplementary material 3.

**Figure 4:**
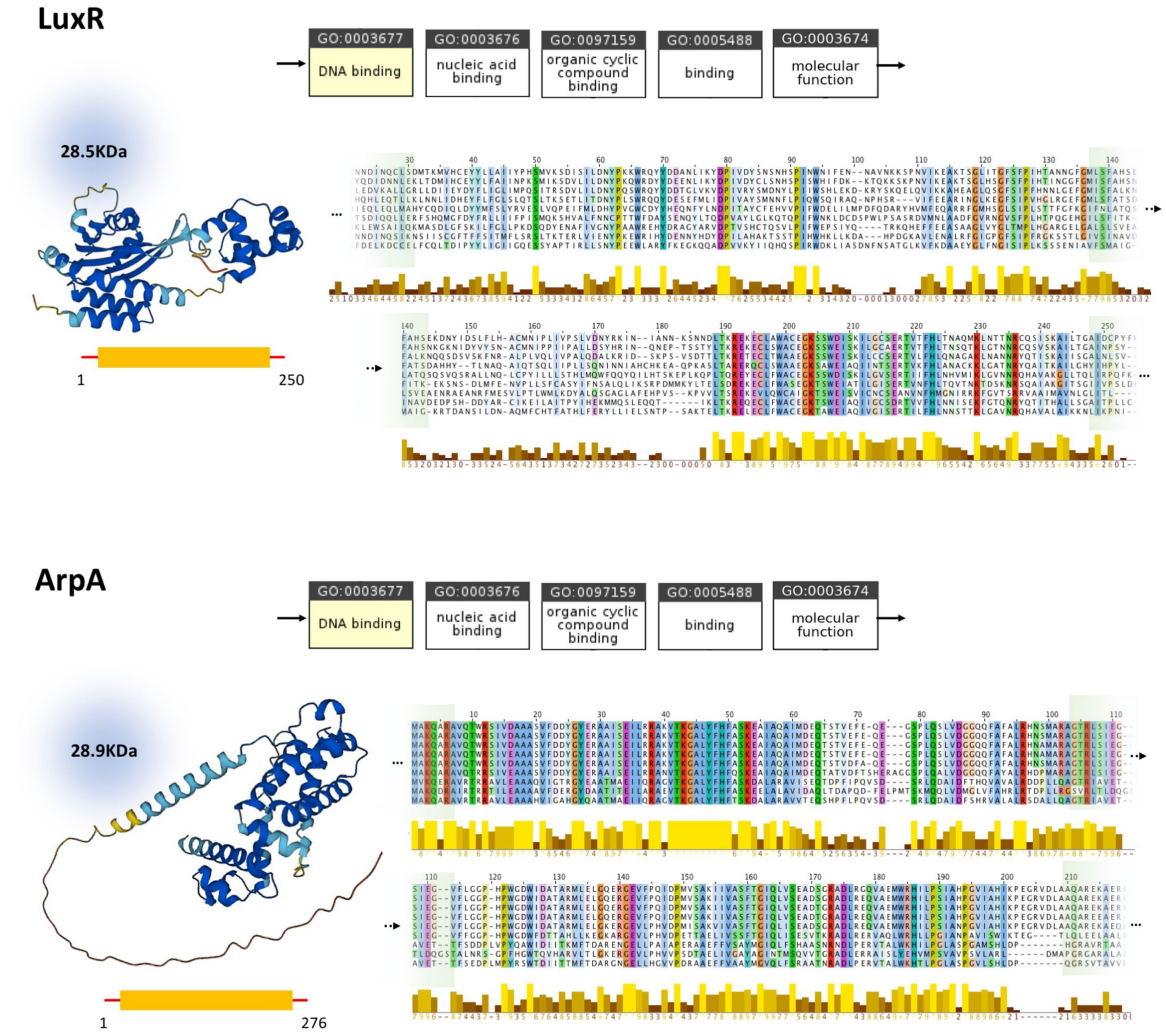
Comparison of LuxR (P12746) and ArpA (Q9ZN78) and homologs. Molecular function ontology, physical properties, and conserved regions of LuxR and ArpA are demonstrated at the top and bottom, respectively. While possessing similar molecular function, molecular weight, and amino acid sequence length, these proteins are different in their three-dimensional structure and the conserved regions obtained by MSA. The full version of MSA is provided in Supplementary material 3.

*In silico* studies investigating the properties of LuxI/LuxR and AfsA/ArpA protein families and their conserved regions, are conducted to assess the status of homology between these protein families. Despite the analogous function of LuxI and AfsA and that of LuxR and ArpA, their close homology could be ruled out based on our current knowledge (Figure 3). According to the ontology of LuxI and AfsA, both enzymes similarly possess transferase activity. Considering the enzymes’ catalytic reactions, in both reactions, the acyl chain originating from the fatty acid-[ACP] is transferred to the other substrate. However, the substrates of LuxI and AfsA are different, as the former is S-adenosyl-L-methionine and the latter is dihydroxyacetone phosphate. Consequently, these enzymes produce different products. AHLs are the product of LuxI, and AfsA produces GBLs. Moreover, the enzymatic reaction of lactone ring formation differs in both enzyme families. The lactone ring in AHLs is formed within the S-adenosyl-L-methionine, and then the fatty acid is bound to the ring acting as the acyl chain, not participating in the lactone ring formation, whereas the reaction of dihydroxyacetone phosphate and fatty acid-[ACP] leads to the formation of the lactone ring in GBLs. In addition, the structure and sequence of LuxI and AfsA were evaluated to assess the possibility of their homology. The sizes of the LuxI and AfsA are 22 and 32 KDa, respectively, and the length of the amino acid sequences are 193 and 301, respectively, which are significantly different. The three-dimensional structure of LuxI and AfsA, as evident in Figure 3, shows no remarkable similarities. Moreover, MSA is conducted to obtain the conserved amino acids of the LuxI and AfsA enzyme families. No similarity in the conserved amino acids of these enzyme families could be found, ruling out the possibility of common motifs responsible for the production of lactone molecules, according to previous studies (Hanzelka et al., 1997; Hsiao et al., 2007). Regarding the comparison of LuxR and ArpA receptor families, both protein families possess a similar molecular function, amino acid sequence length, and molecular weight; however, they are different in their three-dimensional structure and the conserved residues and regions obtained by MSA (Figure 4).

### 3.3 Potential LuxI and LuxR homologs can be found in *Streptomyces*

The *Streptomyces* non-redundant protein sequence database has been examined for potential homologs of LuxI. Four potential homologs of LuxI were found in *Streptomyces* (GGY17999.1, WP_161252959.1, WP_208615587.1, and KOX59212.1) with an *E*-value of maximum 7e-26 and identity and query coverage of at least 35.03 and 84%, respectively (full data are accessible in Supplementary material 1). The sequence range of these proteins was from 191 to 293 amino acids, similar to that of LuxI (190 amino acids). Upon investigation of their function and conserved regions through Interpro, these proteins are annotated as acyltransferase and possess conserved regions found in autoinducer synthases like LuxI. The *Streptomyces* non-redundant protein sequence database has also been evaluated for the existence of potential LuxR homologs via protein–protein BLAST. In contrast to the limited number of hits in the search for LuxI, BLAST of LuxR against the *Streptomyces* proteins resulted in 153 hits with 109 significant hits (*E*-value<0.01) from 70 unique *Streptomyces* species with identity and query coverage of a maximum of 54.9 and 48% (full data are accessible in Supplementary material 1). These proteins were mostly annotated as “response regulator transcription factor,” “LuxR family regulator,” “DNA-binding response regulator,” and “response regulator.” The sequence range of these proteins was from 66 to 482 amino acids. While some potential LuxI and LuxR homologs were found in some *Streptomyces* species, the proteins were limited to a handful of *Streptomyces* species, despite the high abundance of species in this genus. In addition, species examined in this study (except for LuxR in *S. griseus*) could not be found through this method. Moreover, considering the variations in size of the proteins and their significant difference in size compared to LuxR while demonstrating relatively low query coverage and identity, these results could not be seen as satisfactory. To this end, further studies were conducted using LuxI- and LuxR-specific HMMs.

**Figure 5:**
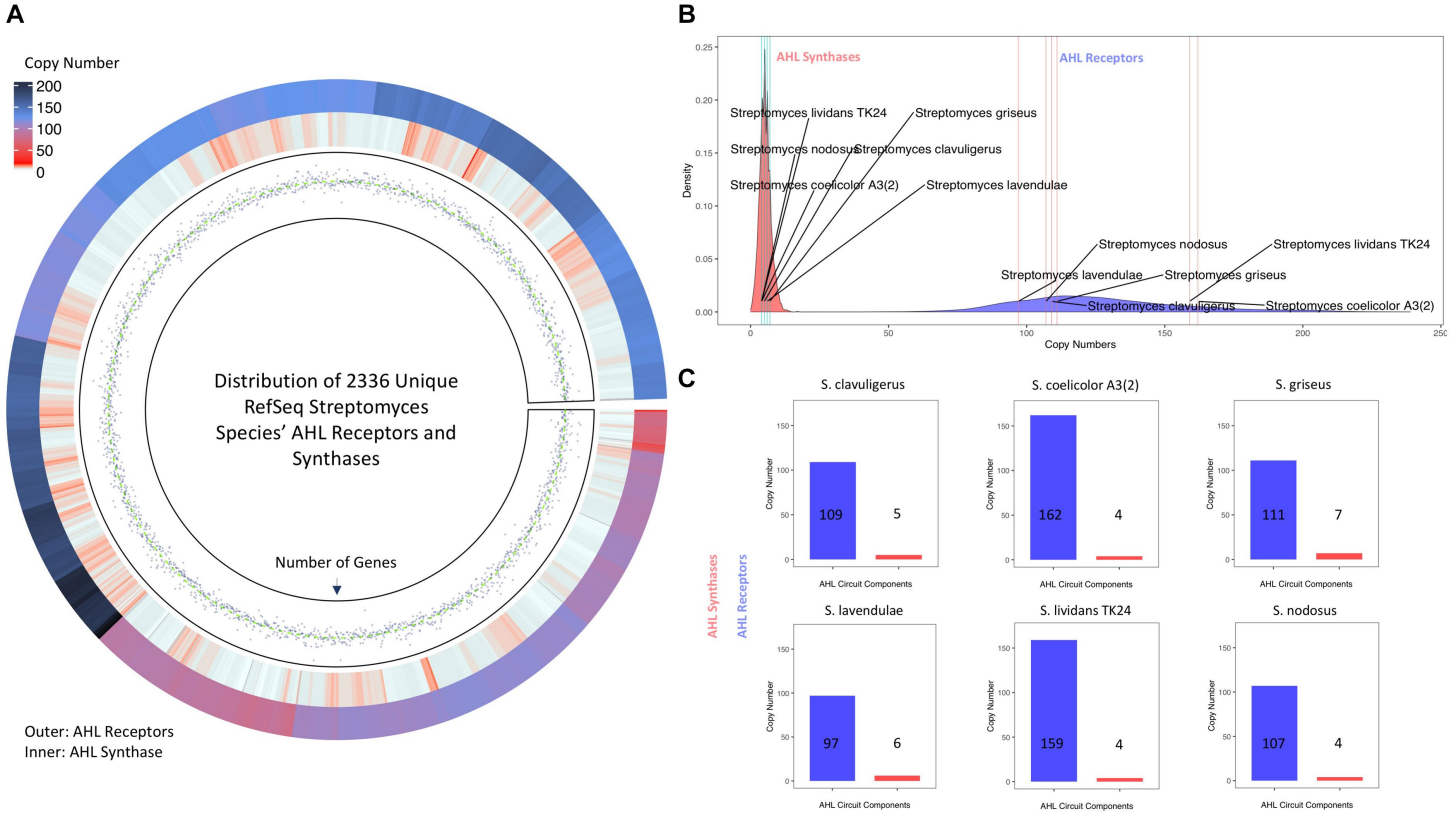
AHL synthases (i.e., LuxI homologs) and AHL receptors (i.e., LuxR homologs) in *Streptomyces* species. **(A)** Circos Heatmap of AHL synthases (inner circle) and AHL receptors (outer circle) found in 2336 unique *Streptomyces* species and points track of number of genes. **(B)** Density plot demonstration of copy numbers of AHL synthases (red) and AHL receptors (blue). The LuxI and LuxR copy numbers of six *Streptomyces* of interest are indexed on each density plot. **(C)** The copy number of AHL synthases (red) and AHL receptors (blue) in the six *Streptomyces* of interest in this study. The data for HMMsearch and protein copy numbers are provided in Supplementary material 1.

Interestingly, screening of the *Streptomyces* proteome for LuxI and LuxR homologs via HMMsearch yielded multiple hits (Figure 5A). Except for *S. abikoensis* (no hits in HMMsearch for LuxI homologs) and *S. mashuensis* (borderline *E*-value of 9.7e-5), all 2,336 unique *Streptomyces* species evaluated in this study possessed at least one potential AHL synthase homologous to LuxI, and all these species harbor at least five AHL receptor homologous to LuxR. The copy number of LuxR homologs was significantly higher than that of LuxI homologs. The maximum copy number of LuxI with 17 copy numbers was found in *S*. sp. *NBC_00051*, and that of LuxR homologs was found in *S. albicerus* with 239 copy numbers. Thirty-eight species possessed only one copy of LuxI, and the minimum copy number of LuxR was five found in *S. cinereus*. LuxI and LuxR homologs were also found in the six *Streptomyces* of interest evaluated for AHLs in this study. It is worth noting that subspecies *S. lividans* TK-64 did not exist in the *Streptomyces* genomes retrieved from NCBI, and other subspecies of *S. lividans* were considered for the analyses. The copy number of AHL synthases (i.e., LuxI homologs) was between four and seven, around the median (*x̃*=5) and average (x̄≈5.3) of these proteins in *Streptomyces* species (Figure 5B). On the other hand, except for *S. nodosus*, *S. griseus*, and *S. clavuligerus*, which resided around the median (*x̃*=123) and average (x̄=126.6) of copy numbers, *S. lividans* TK24 and *S coelicolor* A3(2) possessed more and *S. lavendulae* harbored fewer LuxR homologs (Figures 5B,C).

## 4 Discussion

Gene regulatory circuits are of great interest due to their evolutionary, clinical, and industrial significance. *Streptomyces*, as Gram-positive bacteria, utilize GBL-based gene regulatory circuits to control their noticeable secondary metabolism (e.g., production of antibiotics) and cellular differentiation (Du et al., 2011; Mingyar et al., 2015; Barreales et al., 2020). Notably, GBLs have been only found in *Streptomyces* and are the sole lactone-based autoinducers in these species. On the other hand, AHLs, structurally similar molecules that also take on similar roles, are explicitly attributed to Gram-negative bacteria such as *V. fischeri* (Winson et al., 1995). GBL- and AHL-based gene regulatory circuits also share a common architecture and, along with their components (i.e., synthases and receptors), are considered analogous to each other (Figure 1). This study revealed that while the LuxI and AfsA enzyme families synthesize similar molecules and the LuxR and ArpA receptor families regulate gene expression in response to structurally similar molecules, each protein family has distinct protein structures, amino acid sequences, and conserved regions. Hence, in the absence of an evolutionary relationship between GBLs and AHL circuits, we wondered whether *Streptomyces* possessed AHLs and relevant components.

We found several AHLs produced by *Streptomyces* species, *S. griseus*, *S. lavendulae* FRI-5, *S. clavuligerus*, *S. nodosus*, *S. lividans*, and *S. coelicolor* A3(2), as Gram-positive bacteria, in addition to GBLs. While some AHLs (i.e., m/z 134, 228, 230, 256, and 376) were common between certain *Streptomyces*, each species demonstrated a unique AHL profile. The potential AHL compounds could be seen in four clusters when plotted by their m/z and retention time (Figure 2). Each cluster consists of AHLs from at least four *Streptomyces* species. Moreover, all species possessed at least one compound in the range of m/z 100–200, 200–300, and 300–400, except for *S. lavendulae* FRI-5 and *S. clavuligerus*, lacking any in the first and third range, respectively. The diversity of AHLs from different size ranges may be due to distinct properties of AHLs within each range and their specific physiological roles, which requires *Streptomyces* to possess a set of AHLs with different sizes. For instance, while AHLs with shorter acyl chains (e.g., 3-oxo-C6-HSL) can diffuse passively across the membrane, those with longer and more hydrophobic acyl chains might concentrate in lipid bilayers or require efflux pumps for their accelerated transport (Fuqua et al., 2001; Fuqua and Greenberg, 2002). Moreover, properties of the acyl chain, such as its length, provide specificity to QS systems (Fuqua et al., 2001). Therefore, various AHLs from different ranges might facilitate the regulation of multiple independent gene expressions in a specific species or intra-species and intra-kingdom communications (Shiner et al., 2005; Feng et al., 2014). On the other hand, multiple AHLs and relevant circuitry may also interactively regulate common target genes (Winson et al., 1995).

Based on the mass-spectrometry analyses of standard AHLs conducted here and data available in other reports (Rodrigues et al., 2022), the chemical structure of some AHLs could be predicted. For instance, a molecular ion peak at m/z 256 in *S. lavendulae* FRI-5 and *S. clavuligerus* could be predicted as C10-HSL. Moreover, m/z 228 found in *S. nodosus* and *S. lividans* and m/z 324 in *S. lividans* match C8-HSL and Oxo-C14:1-HSL, respectively. However, further studies such as nuclear magnetic resonance (NMR) analysis are strongly suggested to illuminate the chemical structure of other masses, as they may not consist of a simple acyl chain, such as p-coumaroyl homoserine lactone (Schaefer et al., 2008).

In light of the production of AHLs by *Streptomyces*, these species were evaluated for harboring LuxI and LuxR homologs to further complete the scheme of AHL signaling circuits in *Streptomyces*. The protein–protein BLAST of *V. fischeri*’s LuxI and LuxR against the *Streptomyces* non-redundant protein sequences database resulted in limited homologs of LuxI and LuxR in a handful of *Streptomyces* species. However, the HMMsearch using LuxI- and LuxR-specific HMMs resulted in multiple AHL synthases and receptors homologous to LuxI and LuxR in 2336 unique *Streptomyces*. Previous investigations on the existence of LuxI and LuxR homologs in *Actinobacteria* only resulted in a single potential LuxI homolog in *S. sviceus* (SSEG_02829) (Santos et al., 2012) and some LuxR proteins in a limited number of *Streptomyces* species, including *S. avermitilis* MA-4680, *S. coelicolor* A3(2), and *S. griseus* subsp. griseus NBRC 13350 (Santos et al., 2012). Wet-lab studies on the LuxR family regulator in *Streptomyces* sp. SN593, although with other ligands (*β*-carboline), has proved the functional role of these receptors in gene regulation and secondary metabolism (Panthee et al., 2020).

Interestingly, many *Streptomyces* species were found to carry more than one AHL synthase homologous to LuxI. Similar to most of the *Streptomyces* covered here, the six *Streptomyces* of interest in this study possessed between four and seven LuxI homologs. Harboring multiple AHL synthases could be in line with the ability of these species to produce multiple AHLs of different size ranges. We were not able to establish a positive correlation between the copy number of LuxI homologs and the number of AHLs produced by the six *Streptomyces* of interest, yet we are not surprised due to the imperfection of our methods and the possibility of the role of substrate pools in the combination and abundance of produced AHLs. Regarding the AHL receptors, their high abundance could be interpreted in the context of *Streptomyces*’ rich secondary metabolism and the need for their physiological regulation activities or their inter- and intra-domain interactions and communications with other species. In spite of supporting evidence, it is worth noting that this data should be considered preliminary, and further investigations are suggested to shed light on the origins of these proteins, their structure, the existence of domains of interest, functions, and their physiological and ecological roles.

AHLs have also been reported in a few other Gram-positive bacteria, i.e., *Salinispora* (Bose et al., 2017), *Exiguobacterium* (Biswa and Doble, 2013), and *Bacillus* (de Moura et al., 2021). However, the discovery of AHLs in *Streptomyces* species is accompanied by consequential implications for exploring the evolution of signaling circuits and their role in cellular complexity and multicellularity, in addition to benefiting the industry and the clinic. Moreover, while previous studies only reported a few AHLs (Oxo-C10-HSL and Oxo-C12-HSL in *Salinispora* and Oxo-C8-HSL in *Exiguobacterium*) limited to a single size range, we demonstrated that each *Streptomyces* possesses a handful of AHLs from different size ranges, which is more compatible with the physiological complexity of these organisms. Consequently, these findings expand our understanding of lactone-based gene regulation systems in Gram-positive bacteria, especially *Streptomyces*. Therefore, AHLs might also be involved in regulating secondary metabolites and differentiation in *Streptomyces*, similar to the role of GBLs. While AHLs might regulate specific target genes, they might also interfere with the GBL–receptor interaction by competing with GBLs to occupy the receptor.

Production of AHLs by *Streptomyces species* as Gram-positive bacteria stands against previous knowledge suggesting the exclusivity of AHLs to Gram-negative bacterial species (Azimi et al., 2020). Following our observations, further investigations are required to confirm the existence of AHLs, their synthases and receptors, and the function of AHL-based gene regulation systems in Gram-positive bacteria, specifically in the Actinomycetota phylum and *Streptomyces* species, due to their pharmaceutical and evolutionary significance (Salwan and Sharma, 2020).

## 5 Conclusion

AHLs are generally attributed to Gram-negative bacteria as the mediators of QS. Contrarily, six *Streptomyces* species, *S. griseus*, *S. lavendulae* FRI-5, *S. clavuligerus*, *S. nodosus*, *S. lividans* TK-64, and *S. coelicolor* A3(2), as Gram-positive bacteria, have been found to produce various AHLs, whereas only GBLs have been previously discovered in these species. Furthermore, this study has demonstrated that *Streptomyces* species potentially possess the enzymes necessary for the synthesis of AHLs and the receptors mediating their effects. These results might suggest the potential presence of an additional regulatory and signaling circuit based on AHLs in *Streptomyces*. Furthermore, AHLs and related gene regulatory systems might be more prevalent in the prokaryotic domain than previously assumed, and they might play a role in the regulation of secondary metabolites (e.g., antibiotics), leading to significant industrial and clinical value.

## Data availability statement

The LC-MS/MS raw data presented in the study is deposited in the figshare repository, accession number 10.6084/m9.figshare.25112672. The data used and produced during in-silico studies is provided in the Supplementary material section. Further inquiries can be directed to the corresponding author.

## Author contributions

AS-N: Conceptualization, Formal analysis, Investigation, Methodology, Project administration, Writing – original draft, Writing – review & editing, Validation. ST: Conceptualization, Formal analysis, Investigation, Methodology, Writing – original draft, Data curation, Visualization. GA: Resources, Supervision, Writing – review & editing. JH: Resources, Supervision, Writing – review & editing, Validation.

## Footnotes

^1^ www.geneontology.org

^2^ www.rhea-db.org

^3^ www.ebi.ac.uk

^4^ www.ncbi.nlm.nih.gov/datasets/genome
