# Supplementary material for: Insights into additional lactone-based signaling circuits in Streptomyces: existence of acyl-homoserine lactones and LuxI/LuxR homologs in six Streptomyces species
Source: Front Microbiol. 2024 Feb 8;15:1342637. doi: 10.3389/fmicb.2024.1342637 (PMC10883386; doi:10.3389/fmicb.2024.1342637)

EIC of major peaks from *S. griseus*:

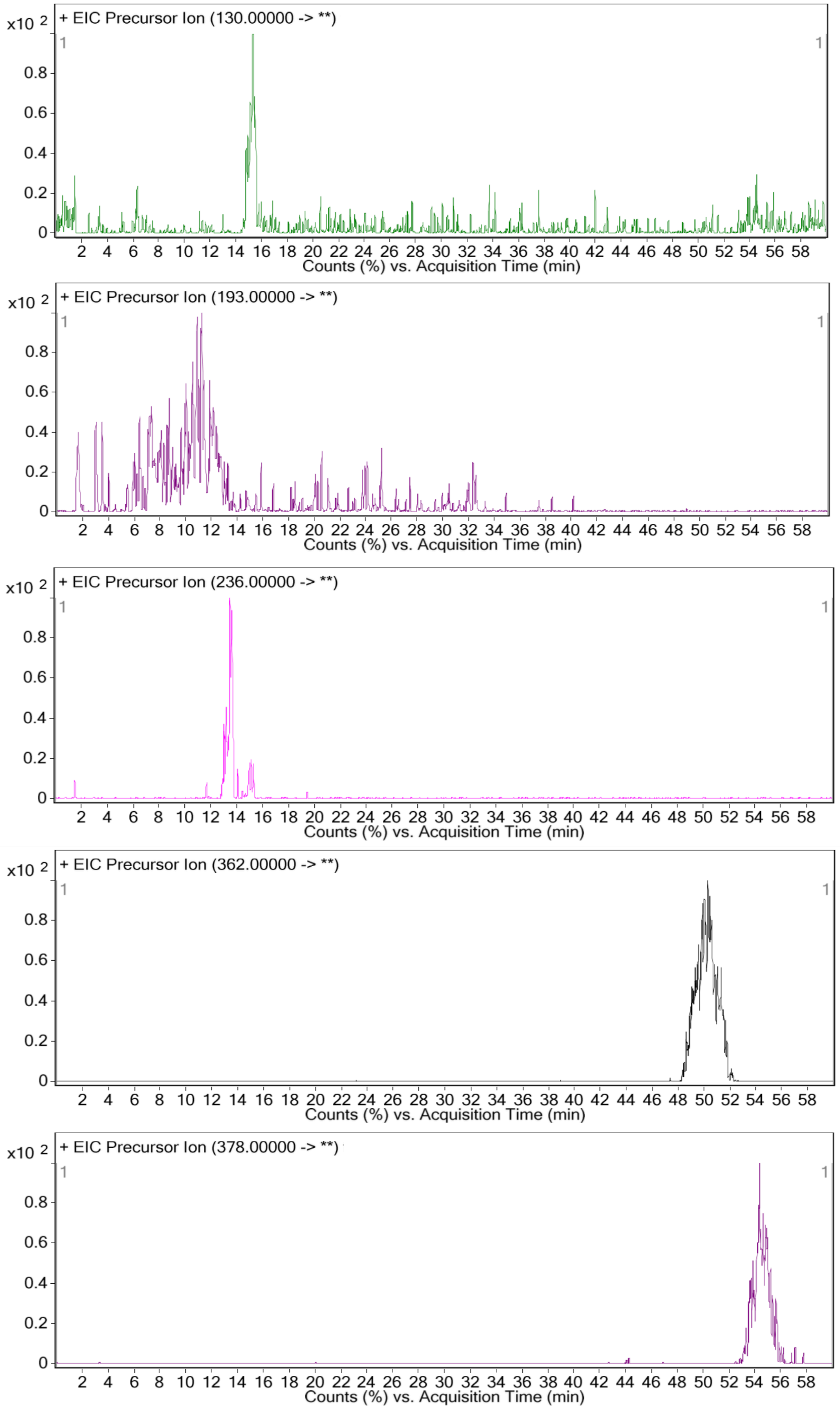

EIC of major peaks from *S. lavendulae* FRI-5:

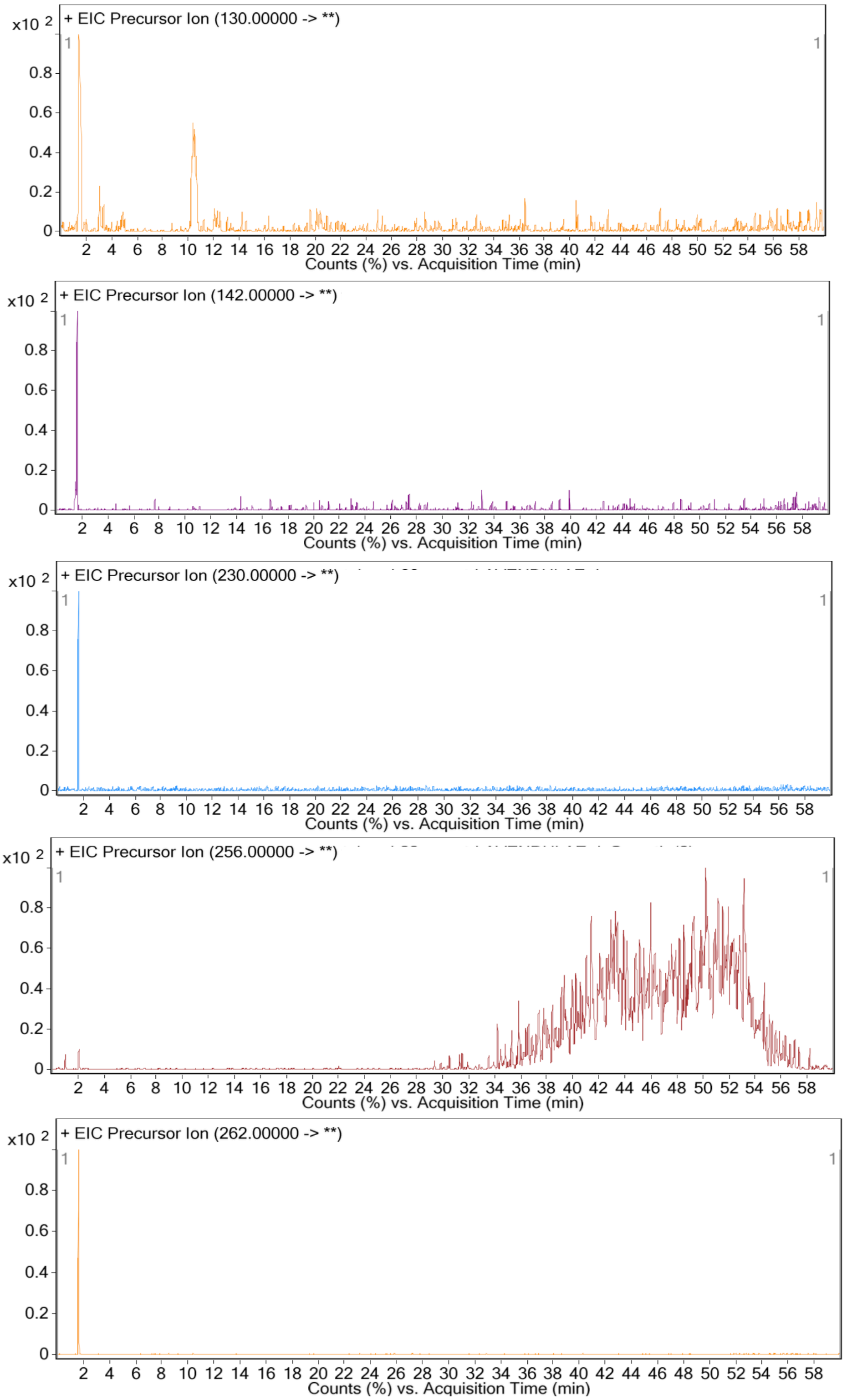

EIC of major peaks from *S. clavuligerus*:

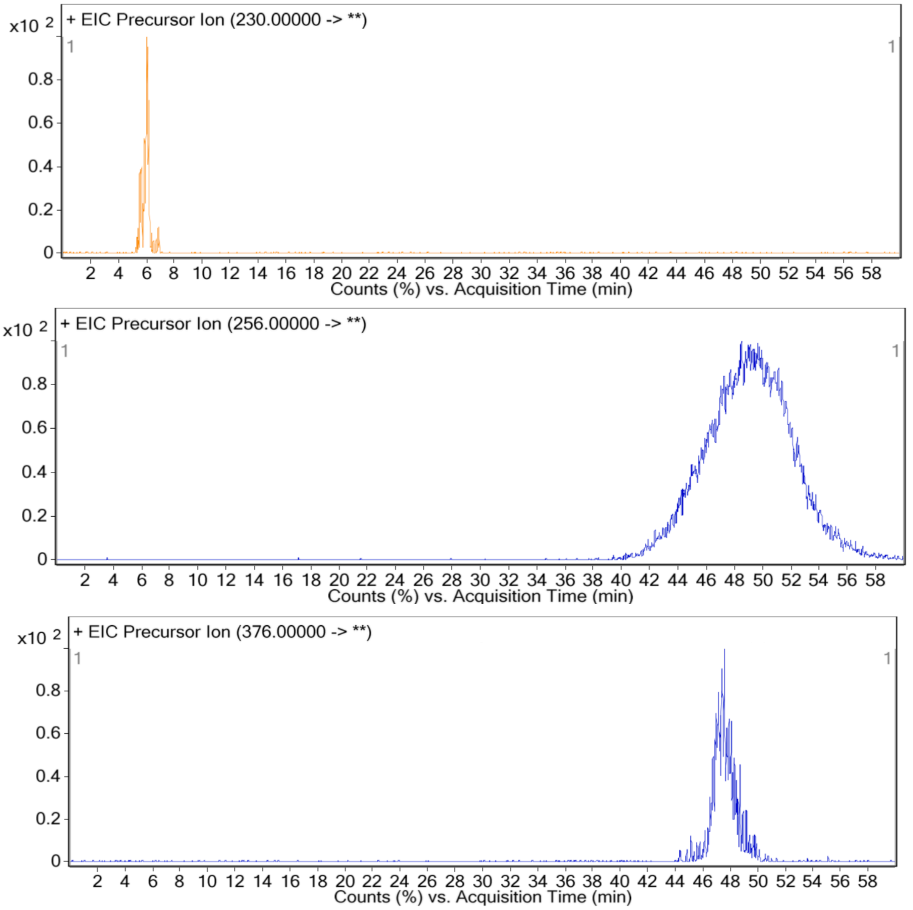

EIC of major peaks from *S. nodosus*:

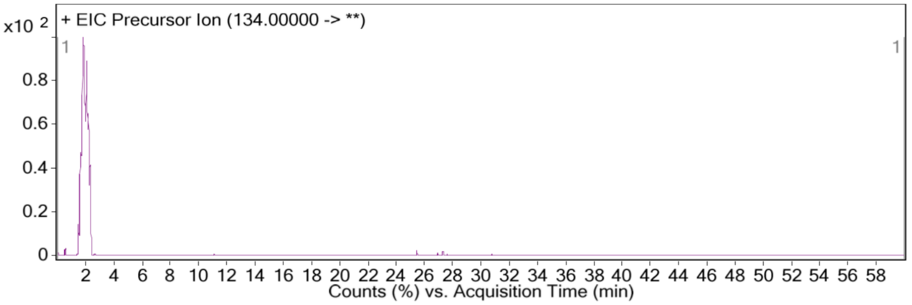

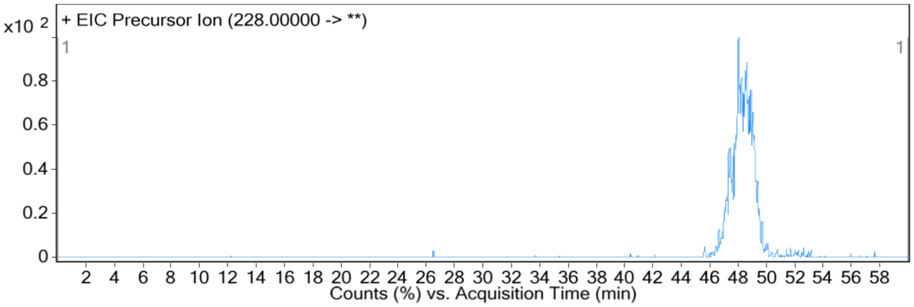

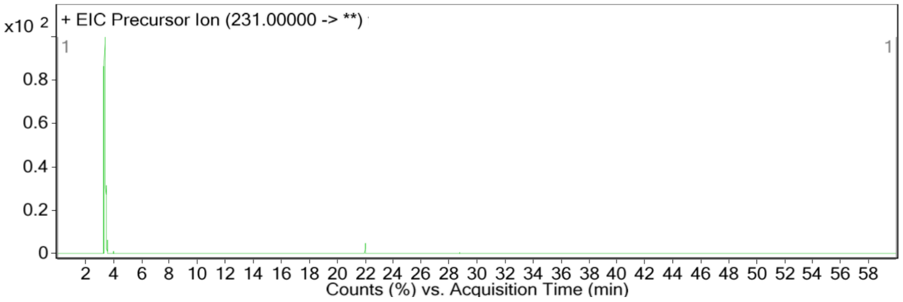

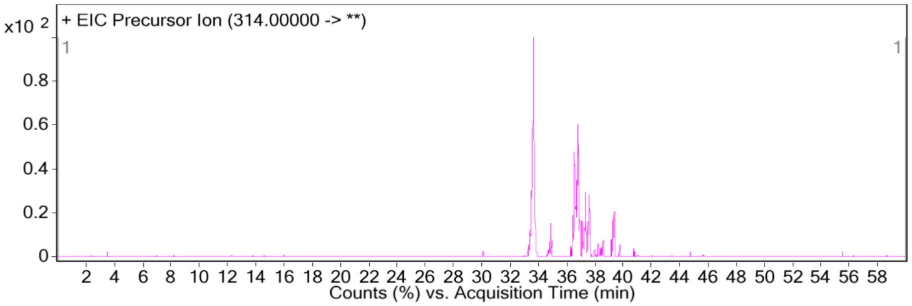

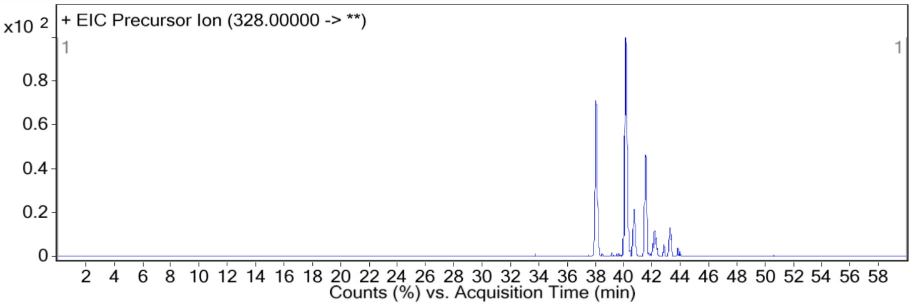

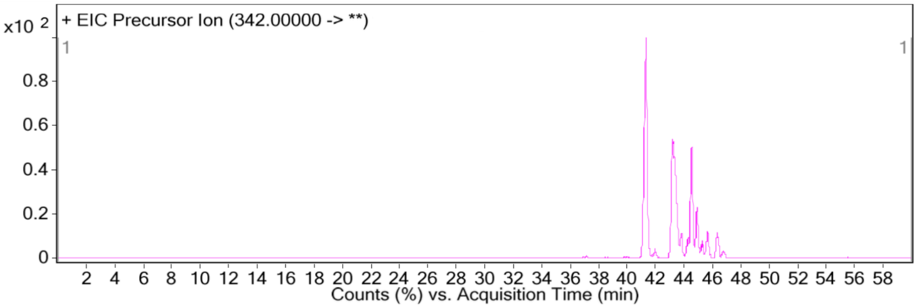

EIC of major peaks from *S. nodosus*:

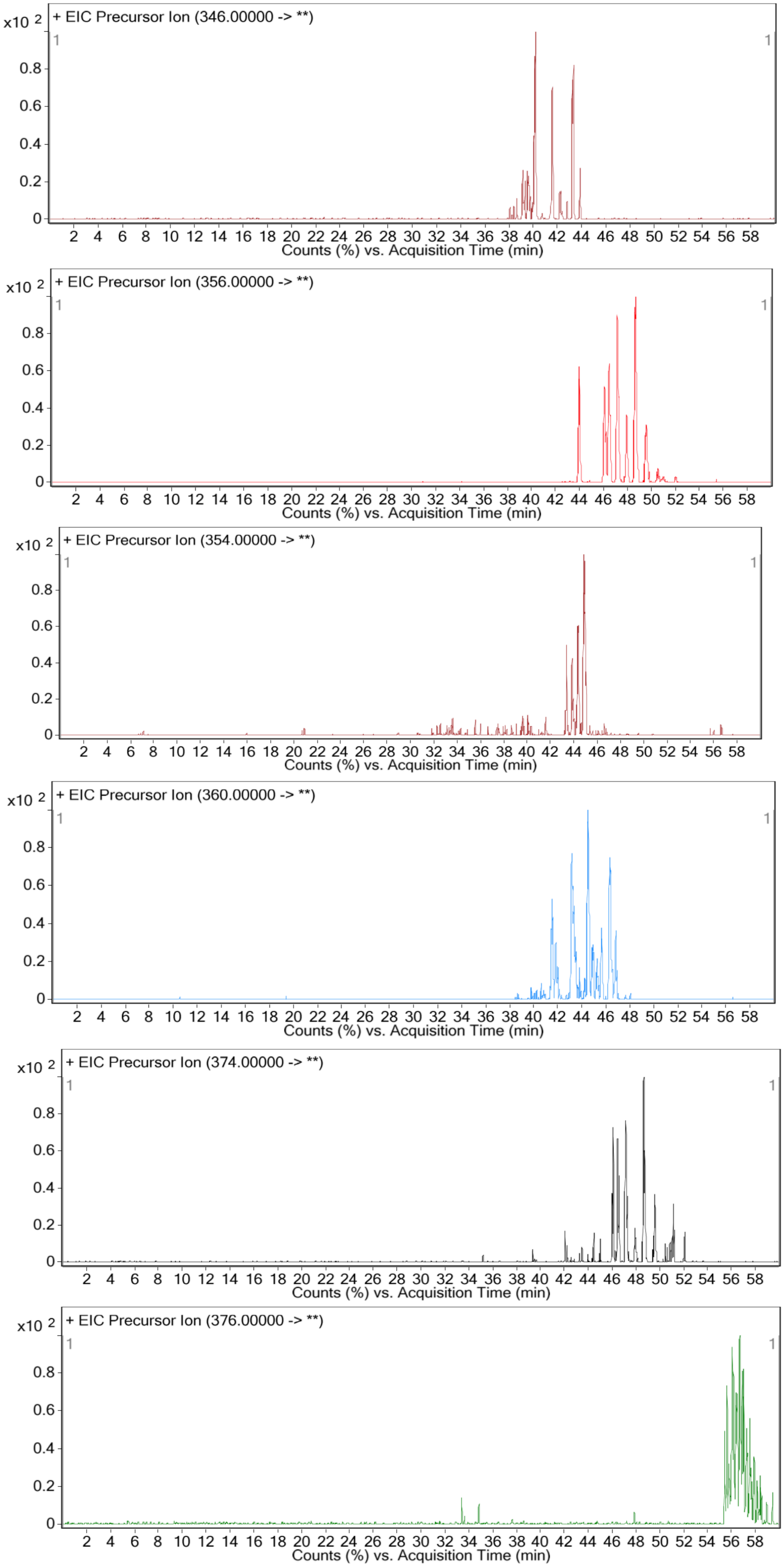

EIC of major peaks from *S. coelicolor* A3(2):

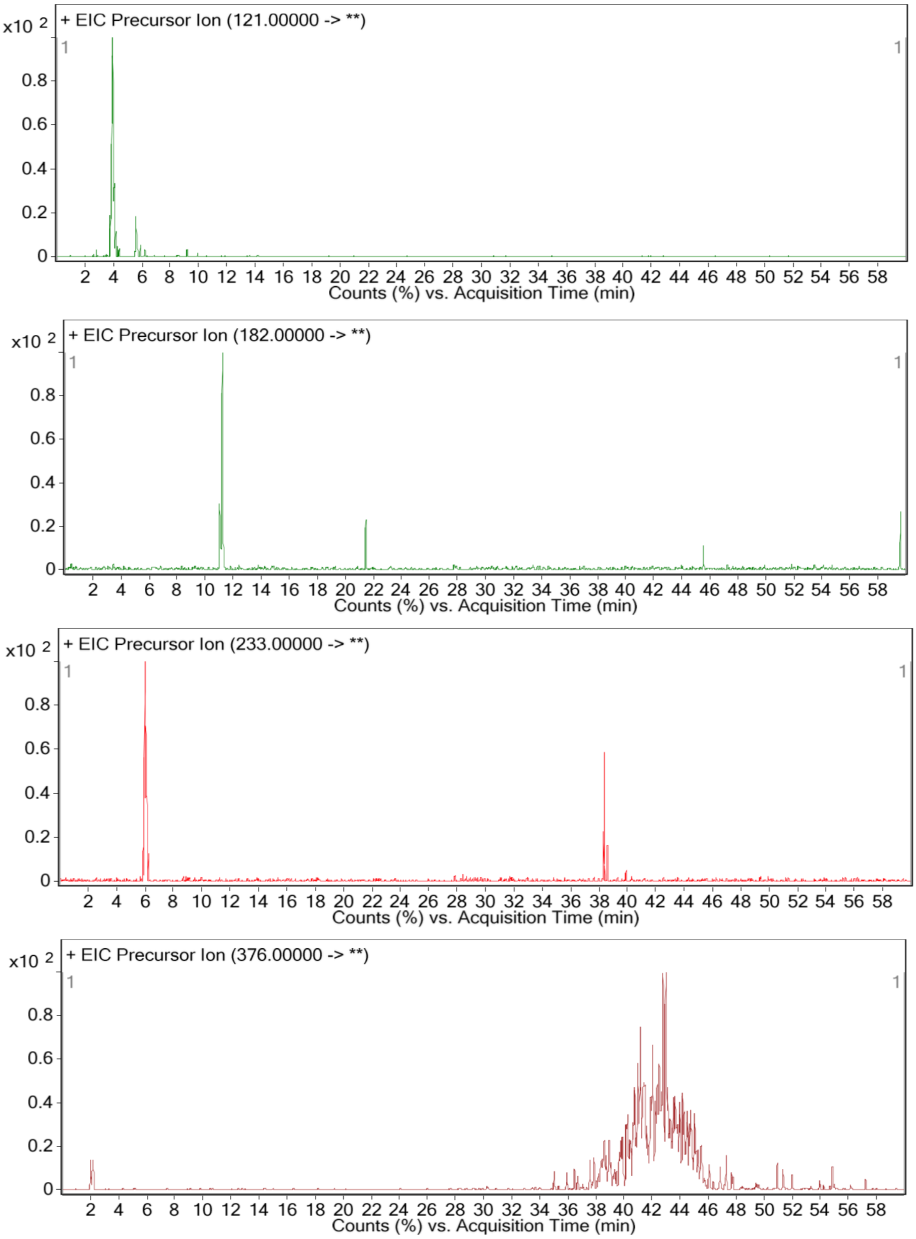

EIC of major peaks from *S. lividans* TK-64:

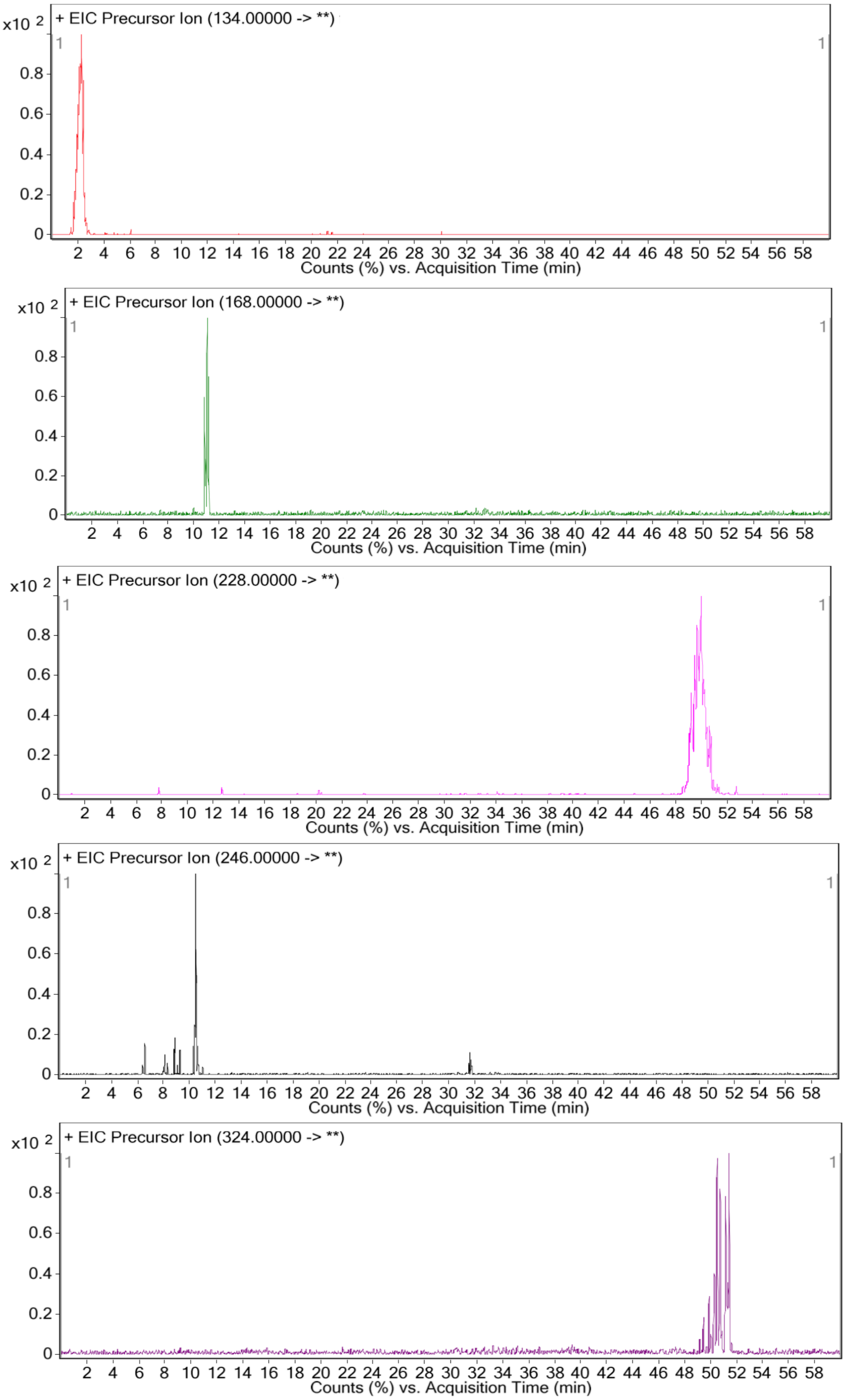

Supplement: Supplementary file 1 [file Data_Sheet_1.zip › Supplementary material 2.PDF]
