# Supplementary figures and images for: Insights into additional lactone-based signaling circuits in Streptomyces: existence of acyl-homoserine lactones and LuxI/LuxR homologs in six Streptomyces species
Source: Front Microbiol. 2024 Feb 8;15:1342637. doi: 10.3389/fmicb.2024.1342637 (PMC10883386; doi:10.3389/fmicb.2024.1342637)

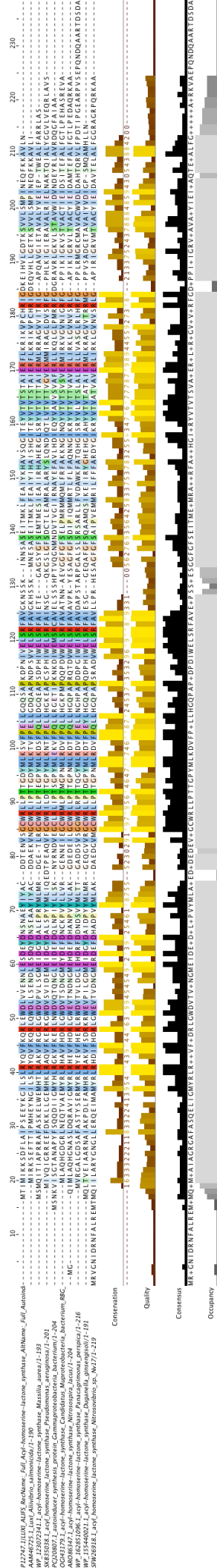

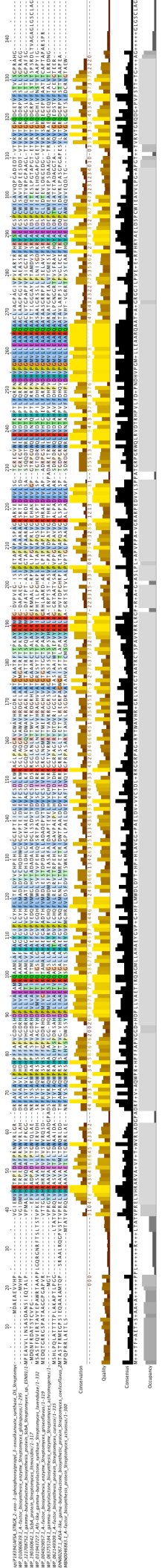

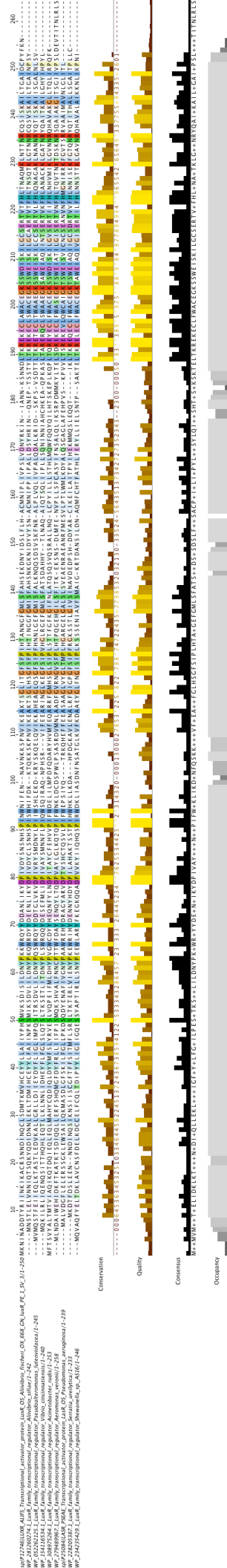

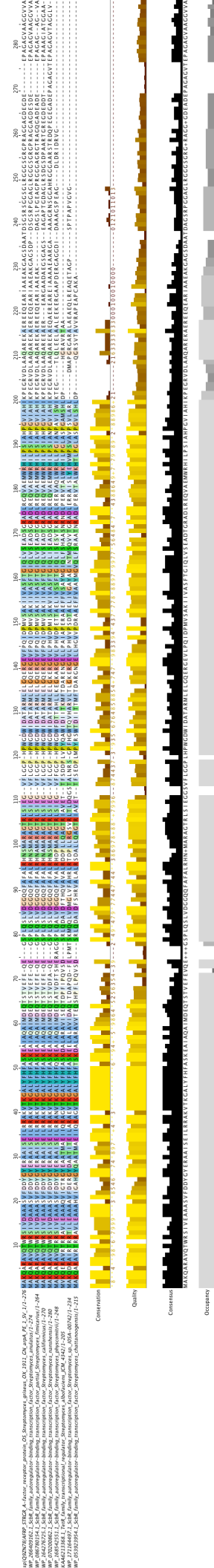

Supplement: Supplementary file 1 [file Data_Sheet_1.zip › Supplementary material 3.PDF]
